# Supplementary material for: Short-chain soluble polyphosphate fertilizers increased soil P availability and mobility by reducing P fixation in two contrasting calcareous soils
Source: PeerJ. 2021 Jul 5;9:e11493. doi: 10.7717/peerj.11493 (PMC8265382; doi:10.7717/peerj.11493)

Figure Captions:

Supplementary Figure 1: Picture of the center lathe machine used in this study and soil sampling method.

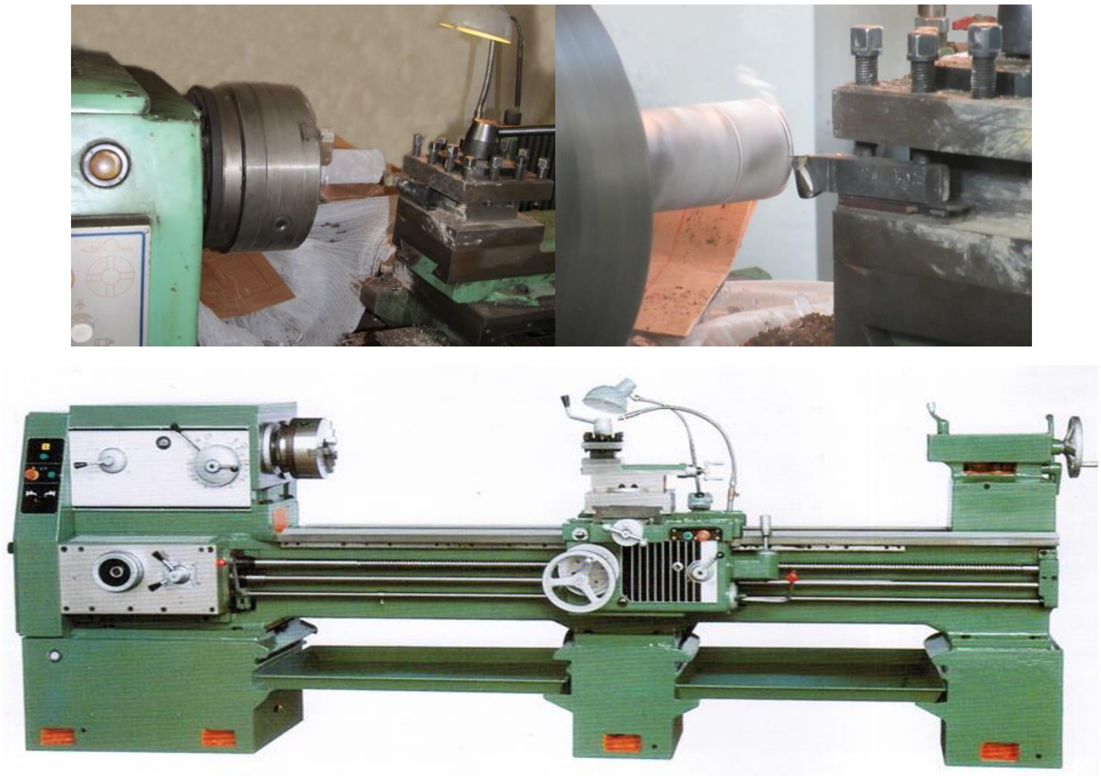

Supplement: Supplemental Information 1 [file peerj-09-11493-s001.pdf]
